# An Outbreak of *Cryptosporidium parvum* across England & Scotland Associated with Consumption of Fresh Pre-Cut Salad Leaves, May 2012

**DOI:** 10.1371/journal.pone.0125955

**Published:** 2015-05-27

**Authors:** Caoimhe McKerr, Goutam K. Adak, Gordon Nichols, Russell Gorton, Rachel M. Chalmers, George Kafatos, Paul Cosford, Andre Charlett, Mark Reacher, Kevin G. Pollock, Claire L. Alexander, Stephen Morton

**Affiliations:** 1 Public Health England, Field Epidemiology Services, London, England, United Kingdom; 2 Department of Gastrointestinal, Emerging & Zoonotic Infections, Public Health England, London, England, United Kingdom; 3 Cryptosporidium Reference Unit, Public Health Wales, Swansea, Wales, United Kingdom; 4 Statistics, Modelling and Economics Department, Public Health England, London, England, United Kingdom; 5 Public Health England, London, England, United Kingdom; 6 Health Protection Scotland, Glasgow, Scotland, United Kingdom; 7 Public Health England Centre, Yorkshire & Humber, England, United Kingdom; 8 Field Epidemiology Training Programme, Public Health England, London, England, United Kingdom; 9 Norwich Medical School, University of East Anglia, Norwich, England, United Kingdom; 10 Faculty of Medicine, University of Thessaly, Larissa, Greece; 11 European Centre for Environment and Human Health, University of Exeter, Exeter, England, United Kingdom; The Australian National University, AUSTRALIA

## Abstract

**Background:**

We report a widespread foodborne outbreak of *Cryptosporidium parvum* in England and Scotland in May 2012. Cases were more common in female adults, and had no history of foreign travel. Over 300 excess cases were identified during the period of the outbreak. Speciation and microbiological typing revealed the outbreak strain to be *C*. *parvum gp60* subtype IIaA15G2R1.

**Methods:**

Hypothesis generation questionnaires were administered and an unmatched case control study was undertaken to test the hypotheses raised. Cases and controls were interviewed by telephone. Controls were selected using sequential digit dialling. Information was gathered on demographics, foods consumed and retailers where foods were purchased.

**Results:**

Seventy-four laboratory confirmed cases and 74 controls were included in analyses. Infection was found to be strongly associated with the consumption of pre-cut mixed salad leaves sold by a single retailer. This is the largest documented outbreak of cryptosporidiosis attributed to a food vehicle.

## Introduction

*Cryptosporidium* is a protozoan parasite, species of which can infect humans and other animals. Transmission can be via the faecal-oral route through the ingestion of oocysts in contaminated food or water, direct contact with animals, or person-person contact [1]. *Cryptosporidium* can be found in drinking water sources; contamination can be through agricultural run-off from grazing land, slurry and discharge of effluents from the treatment of wastewaters and outbreaks have been found to occur following heavy rainfall [2]. Infective stages can remain viable in the environment serving as a reservoir of infection and, in the case of water, as a vehicle of transmission and outbreaks involving drinking or recreational water are often reported [3]. Foodborne outbreaks of cryptosporidiosis are described less frequently and may be difficult to detect [4]. Some food related outbreak reports describe routes of infection as via contamination from water [5], eating raw meat [6] and from infected food handlers at a single event/setting [7]. There are fewer reports of evidence of direct contamination of a food product, particularly one with a wide distribution.

In May 2012 the Health Protection Agency (HPA) in Yorkshire and Humberside (Y&H) identified an increase in the reporting of cryptosporidiosis. Information was sought from other HPA regional epidemiology teams and the HPA Department of Gastrointestinal Emerging and Zoonotic Infections (GEZI). A similar rise was noticed in the North East of England (NE), East Midlands (EM), and West Midlands (WM) and by Health Protection Scotland (HPS). The excess cases were identified by reference genotyping as having *C*. *parvum*. Subsequent analysis of national laboratory reports identified over 300 C*ryptosporidium* cases in excess of numbers expected between weeks 18 and 25 in 2012. Increases in the reporting of cryptosporidiosis were identified from all of the English regions except London during May 2012. A national outbreak control team was convened to design and conduct detailed epidemiological investigations to identify vehicle(s) of infection associated with the outbreak

The following report describes these investigations.

## Materials and Methods

### Statement of ethics

Participants were contacted by trained public health professionals who were specifically aware of the organizations' patient confidentially principles and data protection requirements. Verbal informed consent was sought from all participants at the point of contact and this was recorded on the paper questionnaire and retained. This was explained to all participants. Written consent was not sought due to feasibility and time constraints. As this study concerns an outbreak with direct impact on public health, no approval was required from ethics committee.

### Laboratory report surveillance

Systematic national surveillance of laboratory confirmed infectious intestinal disease, including *Cryptosporidium*, in humans in England and Wales has been established for many years. The system is described elsewhere [8].

### Enhanced surveillance (ES)

Once the increase was noted, enhanced surveillance was introduced in Y&H, NE, EM and WM. A standard questionnaire was augmented to collect detailed descriptive epidemiological data.

Trained public health professionals (Environmental Health Officers) administered these questionnaires to cases either by face to face interviews or over the telephone.

### Hypothesis generation (HG)

A comprehensive hypothesis generation questionnaire was designed. The following types of data were collected: demography; clinical symptoms; contact with animals; recreational exposures; food and drink consumption; food purchasing practices. This was administered by telephone by trained public health professionals to four randomly selected cases of *C*. *parvum* in each of the outbreak regions.

All questionnaires were returned to GEZI for analysis. Exposure frequencies were calculated and those recorded in 75% or more cases were considered as hypotheses for testing in an analytical epidemiological study.

### Case-control study (CC)

In June 2012 a case control study was undertaken to test the hypotheses generated.

### Hypotheses

Main null hypothesis: Being a case of *C*. *parvum* infection > = 16 years of age in the population at risk is **not** associated with general shopping preference with any major food retailer (supermarket chain).

Secondary null hypotheses: Being a case of *C*. *parvum* infection > = 16 years of age in the population at risk is **not** associated with consumption of:

- Bagged salad leaf items;
- Loose salad leaf items;
- Live growing salad leaf items;
- Live growing salad leaf or herb leaf items in pots;
- Live sprouted salad beans items growing in punnets;

in the ten days prior to onset of illness.

It was originally planned to ask about the name of specific ‘own brand’ salad products. When the case control questionnaire was piloted among friends and family of the public health professionals, it became apparent that very few people could recall whether they had bought a specific proprietary product or whether it was a supermarket own brand. However, they could recall whether it was a single salad vegetable such as watercress, spinach or rocket or whether it was a mixed bag salad and could recall which supermarket they had bought these from.

### Case definition

Different case definitions were used for 1. Enhanced surveillance (ES), 2. Hypothesis Generation (HG) and 3. Case-control (CC) parts of the investigation. These were different because they were conducted at different times and when changing information was available. All three restricted investigation to confirmed *Cryptosporidium* cases aged 16 years or more that were resident in Y&H, NE, EM or WM, although CC also included Scottish cases. Exclusions included any patients with a species other than *C*. *parvum*, known secondary cases, people who had recently travelled abroad, or cases known to be part of another cluster of cases. For the ES cases with an onset date on or after 11th May 2012 were included, for the HG cases were chosen with an onset between 11th and 16th May 2012, and for the CC a specimen date of between 11th and 31st May 2012. The HG and CC were only conducted on *C*. *parvum* cases. The CC study did not use patients who had been interviewed previously for the HG.

### Subject recruitment

The Cryptosporidium Reference Unit (CRU) provided health protection specialists in the four English regions with the complete list of cases of PCR-confirmed *C*. *parvum* that conformed to the case definition. The list was ordered by specimen date and a proportionate and systematic sample of cases taken by each area from its own list. A list for Scottish cases was similarly provided by the Scottish Parasite Diagnostic and Reference Laboratory (SPDRL).

One control per case was identified using the systematic digit dialling process after the eligible case had completed a questionnaire, using the land line number of each eligible case interviewed as a starting point. Controls were defined as an adult (16 years or over) resident of the appropriate region identified through the systematic digit dialling process, who had not had vomiting and/or diarrhoea, any foreign travel, or any close contact with a case of diarrhoea, in the ten days prior to the interview.

### Sample size

A sample size of 56 *C*. *parvum* cases and 56 unmatched controls was calculated to distinguish a significant odds ratio of 4 or more for shopping preference at the risk retailer by the case group, compared to control group, for 15% to 65% shopping exposure by the control group.

### Interviewing

Study participants were interviewed via telephone by a trained team of regionally based health protection specialists. Each case plus its associated control was interviewed by the same interviewer wherever possible.

### Data entry and analysis

Data were entered into EpiData Entry (Epidata DK. Denmark, EpiData Association, 2000–2010). Files were inputted in each individual outbreak region, using double data entry and validation techniques. Data were exported into Stata for analyses (StataCorp. Stata statistical software: release 12.1. College Station, USA).

Odds ratios (OR) and P-values (Pearson chi-square) for each exposure were calculated. Individual food exposures and combined exposures to any food items bought from different specified retailers were examined, adjusted for age and sex.

Exposures with an estimated odds ratio in excess of 1 and a p-value of 0.2 or less were included in a multivariable logistic regression model.

All models were adjusted for age and sex.

When detailed microbiological typing was available the analysis was repeated for the most common *gp60* subtype and for other *gp60* subtypes remaining.

### Microbiological typing

All *Cryptosporidium*-positive stools received by the CRU and SPDRL were typed to the species-level in the first instance by real-time PCR incorporating *C*. *parvum*-specific primers and probes [9].

1. All *C*. *parvum* isolates were subtyped by sequencing the *gp60* gene [10, 11, 12] in order to identify if the increase was due to a particular *gp60* subtype.

The outbreak was first detected around 25th May. It became apparent that the exposure was likely to have occurred at the beginning of May and that from the outset all the contaminated product was likely to have been consumed by 25th May. The decision not to sample early suspected foods from Supermarket A was made with input from the Food Standards Agency who had discussions with this supermarket and identified that they sold over 60 different salad products. It was therefore thought to be pointless to sample, because the exact product to sample was unclear, the contaminated product was not available and the methods for sampling for *Cryptosporidium* were complex. Furthermore, at the time there was no standard method for the detection of *Cryptosporidium* in any food item.

## Results

### Laboratory report surveillance

**Fig 1:**
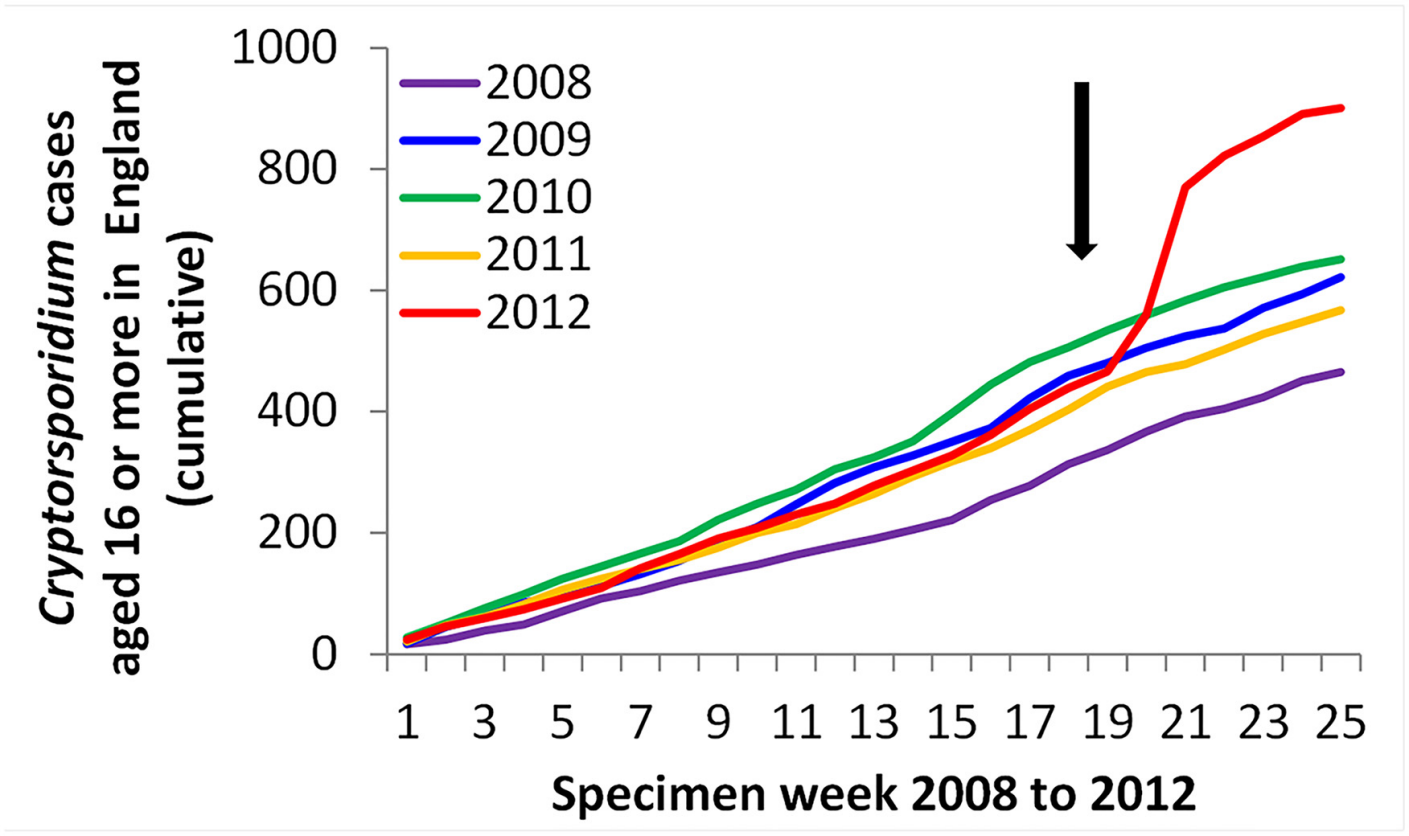
Cumulative count of cases of *Cryptosporidium* sp reported to national surveillance, weeks 1–25 inclusive, England & Wales, 2005–2012.

**Fig 2:**
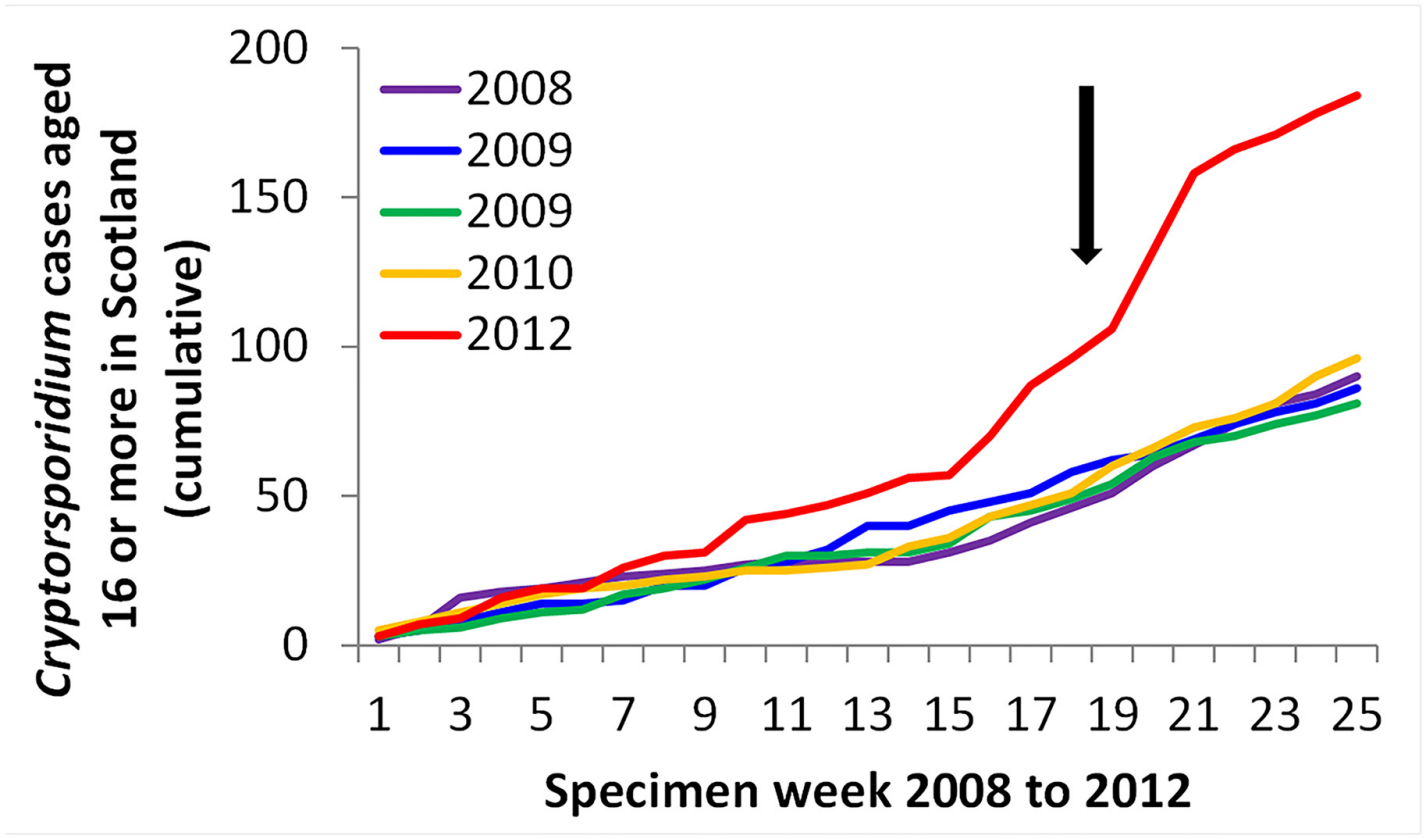
Cumulative count of cases of *Cryptosporidium* sp reported to national surveillance, weeks 1–25 inclusive, Scotland, 2005–2012.

There were marked increases in laboratory reports of *Cryptosporidium spp*. received by national surveillance for Scotland, Wales and each of the English regions, starting in week 19 (week commencing 7th May 2012) in the reporting of *Cryptosporidium spp* in individuals aged 16 years or older in both Scotland and England & Wales (Figs 1 and 2). The Scottish cases increased earlier in 2012 than the English cases, although this earlier increase was not thought to be from the same source. This pattern was not seen in previous years or during 2012 for children aged below 16 years. Case numbers began to decline towards the end of the month, with peaks on the 21st to 22nd May 2012. The peak was in week 21 when 252 cases were reported.

There were 648 cases reported in weeks 19–25 in England & Wales (45% of the year’s cumulative total), 462 of which (71%) were in over 16 year olds. In Scotland, there were 134 cases reported in weeks 19–25 (44% of the year’s cumulative total), 88 of which (66%) were in over 16 year olds.

Our analyses of the age distribution showed that between weeks 19 and 25 of 2012 there was an increase in the proportion of cases in individuals above the age of 16 years. (Table 1) Thus the increase in the reporting of cases during this period was mainly attributable to a sharp increase in laboratory reports of *Cryptosporidium* in older teenagers and adults.

**Table 1:** Range and median age of cases of *Cryptosporidium* spp. reported to national surveillance in weeks 19–25 and in the 4 outbreak regions (Yorkshire and Humberside, North East, East Midlands, and West Midlands), 2007–11 and 2012.

		2007–11	2012
**Male**	Range	0–81	0–90
	Median	11	26
**Female**	Range	0–85	0–95
	Median	21	32
**Total**	Range	0–85	0–95
	Median	18	30

### Enhanced surveillance

**Fig 3:**
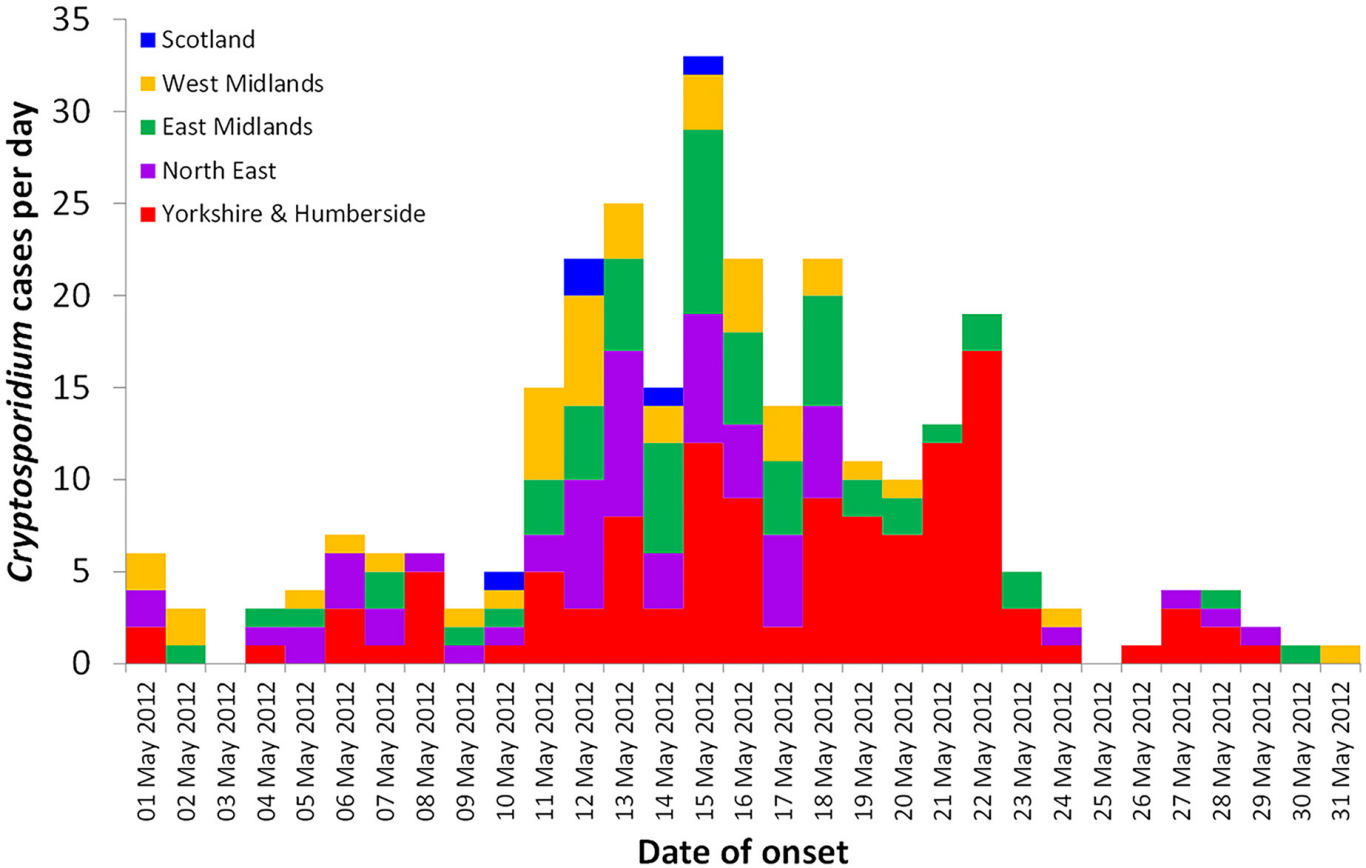
Count of cases of *Cryptosporidium* sp reported to national surveillance by date of onset of illness, May 2012, England regions and Scotland.

Fig 3 shows the epidemic curve for each Health Protection Unit in the four English Regions affected and for Scotland, based on onset date.

### Hypothesis generation

Sixteen questionnaires were administered and returned, four from each region in the outbreak. Preliminary results indicated no link with private water supplies or bottled water/dispensers or animal contact. The following exposures were reported in 75% or more of the cases interviewed, eating salad (n = 16), mixed leaf salad (n = 13), tomatoes (n = 14), cucumber (n = 12), lettuce (n = 12), milk (n = 14), yoghurts (n = 12), bananas (n = 12), raw beef handled in the household (n = 12), preparing raw ingredients (n = 13) and purchasing foods from one particular supermarket chain (Supermarket A) (n = 14).

Milk, yoghurt and beef were discounted because different producers/brands were reported during hypothesis generation, and the items were considered to have come from many different sources, but were still tested in the case control study. Further interrogation of cases’ responses led to the null hypotheses that illness was not associated with any one major food retailer (supermarket chain), or various fresh salad items, including leaves.

### Case control study

Seventy four laboratory-confirmed *C*. *parvum* cases and 74 controls were included for analysis in the study. Cases were generally younger than controls with 60 (81%) cases being under 50 years-old compared to 22 (30%) controls (p<0.001) (Table 2).

**Table 2:** Number and percentage of *C*. *parvum* cases and controls in each age group (years) participating in the case control study, 2012.

	level	control	case	total
age group (years)	20–29	4 (5%)	21 (28%)	25 (17%)
	30–39	10 (14%)	21 (28%)	31 (21%)
	40–49	8 (11%)	18 (24%)	26 (18%)
	50–59	8 (11%)	8 (11%)	16 (11%)
	60–69	26 (35%)	2 (3%)	28 (19%)
	70+	18 (24%)	4 (5%)	22 (15%)
total		74	74	148

Forty-eight (64%) of the cases and 35 (47%) of the controls were female (p = 0.04).

Single variable analysis results for combined exposures bought from any retailer suggested associations between illness and eating rocket, spinach, water cress, mixed leaves, cherry tomatoes, yoghurt, and handling raw beef / eating beef (Table 3).

**Table 3:** Case-control single variable analysis results (adjusted for age and gender) for combined exposures to salad vegetables bought from any Supermarket in the previous 10 days.

	status			single variable analysis		
grouped exposures	level†	control	case	OR	95% CI	p-value
any salad vegetables	no	4	2	baseline		
(148)	yes	70	72	1.92	(0.21, 17.41)	0.561
any cucumber	no	33	28	baseline		
(148)	yes	41	46	0.85	(0.38, 1.9)	0.691
any lettuce	no	23	22	baseline		
(148)	yes	51	52	0.90	(0.39, 2.06)	0.798
any rocket	no	59	44	baseline		
(147)	yes	14	30	2.28	(0.97, 5.37)	0.060
any spinach	no	65	47	baseline		
(147)	yes	8	27	3.12	(1.15, 8.42)	0.025
any water cress	no	62	55	baseline		
(147)	yes	11	19	2.01	(0.75, 5.41)	0.166
any mixed leaves	no	41	21	baseline		
(147)	yes	32	53	2.36	(1.03, 5.37)	0.041
any beef tomatoes	no	63	69	baseline		
(146)	yes	10	4	0.27	(0.06, 1.16)	0.079
any cherry tomatoes	no	38	20	baseline		
(147)	yes	35	54	2.25	(1, 5.06)	0.050
any plum tomatoes	no	60	62	baseline		
(148)	yes	14	12	0.76	(0.29, 2.03)	0.587
any salad tomatoes	no	35	53	baseline		
(148)	yes	39	21	0.32	(0.14, 0.73)	0.007
any pre-cut lettuce	no	36	24	baseline		
(121)	yes	24	37	1.02	(0.41, 2.52)	0.971
any pre-cut rocket	no	37	28	baseline		
(105)	yes	12	28	2.09	(0.8, 5.46)	0.134
any pre-cut spinach	no	41	30	baseline		
(103)	yes	6	26	3.39	(1.04, 11.02)	0.042
any pre-cut water cress	no	41	36	baseline		
(99)	yes	7	15	2.00	(0.61, 6.56)	0.253
any pre-cut mixed leaves	no	25	12	baseline		
(116)	yes	27	52	2.03	(0.71, 5.83)	0.189
any bananas	no	19	26	baseline		
(148)	yes	55	48	0.91	(0.39, 2.09)	0.818
any cold cows’ milk	no	38	45	baseline		
(147)	yes	36	28	0.56	(0.25, 1.26)	0.163
any yoghurt	no	40	27	baseline		
(148)	yes	34	47	1.97	(0.89, 4.35)	0.093
any handling raw beef / eating beef	no	40	25	baseline		
(147)	yes	33	49	1.88	(0.85, 4.15)	0.119
Supermarket D—any food item	no	58	54	baseline		
(141)	yes	12	17	1.97	(0.69, 5.62)	0.205
Supermarket C—any food item	no	50	46	baseline		
(141)	yes	20	25	0.66	(0.27, 1.64)	0.372
Supermarket E—any food item	no	47	48	baseline		
(140)	yes	22	23	0.57	(0.23, 1.42)	0.229
Supermarket F—any food item	no	67	64	baseline		
(140)	yes	3	6	2.87	(0.53, 15.60)	0.223
Supermarket G—any food item	no	62	55	baseline		
(140)	yes	8	15	1.57	(0.51, 4.81)	0.428
Supermarket A—any food item	no	47	18	baseline		
(146)	yes	26	55	8.73	(3.40, 22.38)	<0.001
Supermarket I—any food item	no	49	49	baseline		
(142)	yes	20	24	1.21	(0.52, 2.82)	0.656
Supermarket B—any food item	no	44	41	baseline		
(141)	yes	26	30	0.62	(0.27, 1.43)	0.262
Supermarket J—any food item	no	61	67	baseline		
(140)	yes	8	4	0.53	(0.13, 2.20)	0.380
Other shops—any food item	no	13	16	baseline		
(84)	yes	29	26	1.27	(0.43, 3.74)	0.668

^†^ The level refers to whether the patient responded with a yes or no to the exposure question.

Single variable analysis results for exposures by retailer showed evidence of association between illness and pre-cut salad items bought from Supermarket A, B and C. 72% of cases reported having consumed salad items bought from Supermarket A in the 10 days before the onset of their illness.

Variables eligible for inclusion in the multivariable regression model were: pre-cut rocket bought from any retailer, pre-cut spinach bought from any retailer, water cress bought from any retailer, pre-cut mixed salad leaves bought from any retailer, cherry tomatoes bought from any retailer. Spinach bought from any retailer was the only combined food variable significantly associated with being a case.

In the multivariable logistic regression analysis pre-cut mixed salad leaves bought from Supermarket A, plum tomatoes from Supermarket B, pre-cut spinach from Supermarket C and milk bought from Supermarket A were significantly associated with illness. (Table 4)

**Table 4:** Multivariable logistic regression analysis adjusting for gender and age.

Exposure	Level†	No (%) cases' exposure	OR	95% CI	p-value
Pre-cut mixed leaves from Supermarket A	no	32 (48.5)	baseline		
	yes	34 (51.5)	7.71	(2.40, 24.78)	<0.001
Plum tomatoes bought from Supermarket B	no	68 (91.9)	baseline		
	yes	6 (0.08)	10.71	(1.27,.)	0.017
Pre-cut spinach bought from Supermarket C	no	65 (89.0)	baseline		
	yes	8 (11.0)	11.27	(1.40,.)	0.028
Milk bought from Supermarket A	no	58 (79.5)	baseline		
	yes	15 (20.5)	4.33	(0.69, 27.05)	0.096

^†^ The level refers to whether the patient responded with a yes or no to the exposure question.

6 out of 74 cases (8%) bought plum tomatoes from Supermarket B and 11% (8 out of 73 cases) bought pre-cut spinach from Supermarket C. Greater proportions bought milk at 24% (15 out of 63) and pre-cut mixed leaves at 52% (34 out of 66) from Supermarket A.

### Microbiology

Of the 648 cases of cryptosporidiosis reported in England and Wales in weeks 19–25, 312 cases were reported in weeks 20–22 and 289 (93%) of these were identified as *C*. *parvum*. The SPDRL speciated 24/134 isolates received from Scottish laboratories in the same time and 20 of these (87%) were identified as *C*. *parvum*.

The excess in adult cases in *C*. *parvum* was most noticeable in weeks 20–22, whereas *C*. *hominis* cases did not change during that time period. Thus there was no evidence for a mixed *C*. *hominis* and *C*. *parvum* outbreak.

#### GP60 analysis

Of 182 confirmed *C*. *parvum* cases in the affected English regions and Scotland that were sequenced at the *gp60* gene, 142 (78%) were subtype IIaA15G2R1. A total of 63/75 cases recruited to the case-control study were of this predominant subtype.

In multivariable analysis there was a there was strong association with consumption of spinach (pre-cut and whole) bought from Supermarket A, with a higher odds ratio than in the ordinary multivariable analysis. There were also positive associations between eating plum tomatoes bought from Supermarket B, pre-cut spinach bought from Supermarket C, and with milk also bought from Supermarket A. None of these exposures were associated with the other *C*. *parvum* subtypes (Table 5).

**Table 5:** Multivariable logistic regression analysis adjusting for gender and age, by *C*. *parvum gp60* subtype.

Exposure	Level†	OR	95% CI	p-value
***gp60 subtype IIaA15G2R1***				
Pre-cut mixed leaves from Supermarket A	no	baseline		
	yes	11.93	(3.29, 43.22)	<0.001
Plum tomatoes bought from Supermarket B	no	baseline		
	yes	12.96	(1.27,.)	0.03
Pre-cut spinach bought from Supermarket C	no	baseline		
	yes	11.12	(1.28,.)	0.028
Milk bought from Supermarket A	no	baseline		
	yes	5.37	(0.75, 38.65)	0.095
***other gp60 subtyping***				
Pre-cut mixed leaves from Supermarket A	no	baseline		
	yes	1.05	(0.10, 11.21)	0.968
Plum tomatoes bought from Supermarket B	no	baseline		
	yes	1.07	(0,.)	1
Pre-cut spinach bought from Supermarket C	no	baseline		
	yes	1.17	(0,.)	1
Milk bought from Supermarket A	no	baseline		
	yes	2.86	(0.20, 40.85)	0.438

^†^ The level refers to whether the patient responded with a yes or no to the exposure question.

## Discussion

Between weeks 19 and 25, 2012, 648 cases of *Cryptosporidium spp*. were reported across England & Wales and 134 across Scotland with a marked peak between weeks 19 and 21. Cases were predominantly in adults. There was a higher than expected percentage of female cases, although this was not significantly different from the same weeks in previous years. The number of cases reported was greater in some areas than others which may be indicative of differences in distribution of a product. However, we cannot exclude ascertainment differences by region as a contributory factor. The epidemiological investigations support the hypothesis that salad items, particularly from one retailer, were the vehicle for infection.

The most plausible cause of the outbreak from the multivariable logistic regression was pre-cut mixed leaves from Supermarket A, with 51% of interviewees reporting this exposure and a strong odds ratio (7.71 with CI 2.40–24.78) and significance value. The other exposures (Plum tomatoes Supermarket B, Pre-cut spinach Supermarket C, Milk Supermarket A) all represent under 21% of cases, and could be explained by confounding. By this we mean that salad items are more likely to be consumed by people who were eating pre-cut mixed leaves and milk from Supermarket A is more likely to be consumed by people buying pre-cut mixed leaves from Supermarket A. Two of the four exposures showing significance in the model were related to consuming salad vegetables bought pre-cut in sealed bags. This could be indicative of one or more types of salad vegetables having been washed in contaminated water, having been contaminated during handling from the producer or during growing or irrigation.

Our findings are supported by several reports in the literature highlighting contamination of salad items. This has been reported in outbreaks of shiga toxin producing *Escherichia coli* and salmonella from various parts of the World. [13, 14, 15]. There are risks associated with the contamination of fresh produce either directly from animal dung or indirectly by water used for irrigation and washing, and *Cryptosporidium* oocysts can be difficult to remove from leaves [5, 16]. The risks of *Cryptosporidium* transmission from salad products contaminated from irrigation water has previously been described through a survey of fresh salad products [17]. Cryptosporidiosis has previously been linked to raw vegetable consumption in Denmark, Sweden and Finland [18, 19, 20].

The food trace back data in this outbreak revealed that Supermarket A was not the sole recipient of any of the implicated vehicles of infection. There were 61 different salad products supplied by Supermarket A and included leaves from growers in the UK, Spain, Italy and France. It was not possible to identify the exact products people ate or how mixing and batching of produce might have affected the distribution of contaminated salad leaves in the finished bags sold to the public. Confounding cannot be discounted given that salad leaves are often eaten in combination with other items, and recall bias may also affect case and control reports of foods bought i.e. individuals who usually buy their food from other supermarkets omit to mention a one-off purchase from another supermarket. However, food investigations were unable to identify any potential expected confounders and the short duration of the outbreak eliminates suspects such as dressings and oils, which have a longer shelf life. Complexities and limited traceability in identifying patterns of salad vegetable distribution may hinder further investigation of the ultimate sources of these kinds of outbreaks despite thorough investigation [9, 21].

Fresh produce remains a risk for gastro-intestinal illness and recommendations for the investigation of future outbreaks of this nature include coordination between epidemiological and food investigations so that inquiries can be expedited and preliminary findings reported and acted upon. This has been the predominant approach among the most successful and expedient outbreak investigations. In 2004, Swedish authorities notified the finding of *Salmonella* Thompson in rucola lettuce through the EU Rapid Alert System for Food and Feed, aiding on-going outbreak investigations and supporting the same findings in other countries [10].

UK outbreaks, such as *Salmonella* Bareilly in 2010, and the large outbreak of *Escherichia coli* O104:H4 in Germany in 2011 highlight the importance of trace back investigations which can lead to individual suppliers for recall and investigation [22, 23]. This is the largest outbreak of foodborne cryptosporidiosis reported globally and indicates an increased need for awareness of the mechanisms by which protozoa may be transported on salad leaves and the importance of surveillance for *Cryptosporidium*.

## References

[1] Casemore D (1990) Epidemiological aspects of human cryptosporidiosis. Epidemiol Infect. 104, 1–28 2407541 10.1017/s0950268800054480 PMC2271741

[2] Lake IR, Bentham G, Kovats RS, Nichols GL (2005) Effects of weather and river flow on cryptosporidiosis. J Water Health 469–474. 16459850 10.2166/wh.2005.048

[3] Smith A, Reacher M, Smerdon W, Adak GK, Nichols G, Chalmers RM (2006) Outbreaks of Waterborne Intestinal Disease in England and Wales 1992–2003. Epidemiol Infect.134:1141–1149 16690002 10.1017/S0950268806006406 PMC2870523

[4] Robertson L J, Chalmers RM (2003) Foodborne cryptosporidiosis: is there really more in Nordic countries? Trends in Parasitology (29)1:3 9 10.1016/j.pt.2012.10.003 23146217

[5] Mota A, Mena KD, Soto-Beltran M, Tarwater PM, Chaidez C (2009) Risk assessment of cryptosporidium and giardia in water irrigating fresh produce in Mexico. J Food Prot 72:2184–2188. 19833043 10.4315/0362-028x-72.10.2184

[6] Yoshida H, Matsuo M, Miyoshi T, Uchino K, Nakaguchi H, Fukumoto T, et al (2007) An outbreak of cryptosporidiosis suspected to be related to contaminated food, October 2006, Sakai City, Japan. Jpn J Infect Dis. 60:405–7. 18032847

[7] Quiroz ES, Bern C, MacArthur JR, Xiao L, Fletcher M, Arrowood MJ, et al (2000) An outbreak of cryptosporidiosis linked to a food handler. J Infect Dis. 181:695–700. 10669357 10.1086/315279

[8] Wall PG, de Louvois J, Gilbert RJ, Rowe B (1996) Food poisoning: notifications, laboratory reports and outbreaks—where do the statistics come from and what do they mean? CDR Review 6:93–100. 8680502

[9] Hadfield SJ, Robinson G, Elwin K, Chalmers RM (2011) Detection and differentiation of Cryptosporidium spp. in human clinical samples by use of real-time PCR. Journal of Clinical Microbiology 49(3): 918–924 10.1128/JCM.01733-10 21177904 PMC3067739

[10] Alves M, Xiao L, Sulaiman I, Lal AA, Matos O, Antunes F (2003) Subgenotype analysis of Cryptosporidium isolates from humans, cattle, and zoo ruminants in Portugal. Journal of Clinical Microbiology 41; 2744–7. 12791920 10.1128/JCM.41.6.2744-2747.2003 PMC156540

[11] Sulaiman IM, Hira PR, Zhou L, Al-Ali FM, Al-Shelahi FA, Shweiki HM, et al (2005) Unique endemicity of cryptosporidiosis in children in Kuwait. J Clin Microbiol.;43:2805–9 15956401 10.1128/JCM.43.6.2805-2809.2005 PMC1151898

[12] Nichols GL, Chalmers RM, Hadfield SJ. Molecular Epidemiology of Human Cryptosporidiosis In: Cryptosporidium: parasite and disease. Editors: Cacciò Simone M. & Widmer Giovanni. ISBN: 978-3-7091-1561-9 (Print) 978-3-7091-1562-6 (Online) 2014.

[13] Irvine WN, Gillespie IA, Smyth FB, Rooney PJ, McClenaghan A, Devine MJ, et al; Outbreak Control Team (2009) Investigation of an outbreak of Salmonella enterica serovar Newport infection. Epidemiol Infect. 10;137(10):1449–56. 10.1017/S0950268809002416 Epub 2009 Mar 19. 19296871

[14] Nygård K, Lassen J, Vold L, Andersson Y, Fisher I, et al (2008) Outbreak of Salmonella Thompson infections linked to imported rucola lettuce. Foodborne Pathog Dis. 4;5(2):165–73. 10.1089/fpd.2007.0053 18361685

[15] Slayton RB, Turabelidze G, Bennett SD, Schwensohn CA, Yaffee AQ, et al (2013) Outbreak of Shiga toxin-producing Escherichia coli (STEC) O157:H7 associated with romaine lettuce consumption, 2011. PLoS One. 8(2):e55300 10.1371/journal.pone.0055300 Epub 2013 Feb 4. 23390525 PMC3563629

[16] Macarisin D, Bauchan G, Fayer R (2010) Spinacia oleracea L. leaf stomata harboring Cryptosporidium parvum oocysts: a potential threat to food safety. Appl. Environ. Microbiol. 76, 555–559 10.1128/AEM.02118-09 19933348 PMC2805213

[17] Amorós I, Alonso JL, Cuesta G (2012) Cryptosporidium oocysts and Giardia cysts on salad products irrigated with contaminated water. Journal of Food Protection, 73: 1138–1140. 10.4315/0362-028x-73.6.1138 20537274

[18] Ethelberg S, Lisby M, Vestergaard LS, Enemark HL, Olsen KE, Stensvold CR et al (2009) A foodborne outbreak of Cryptosporidium hominis infection, Epidemiol. Infect. 137(3):348–56 10.1017/S0950268808001817 19134228

[19] Insulander M, de Jong B, Svenungsson B (2008) A food-borne outbreak of cryptosporidiosis among guests and staff at a hotel restaurant in Stockholm county, Sweden, September 2008. Euro Surveill. 13(51):19071 19094915

[20] Pönka A, Kotilainen H, Rimhanen-Finne R, Hokkanen P, Hänninen ML, Kaarna A, et al (2009) A foodborne outbreak due to Cryptosporidium parvum in Helsinki, November 2008. Euro Surveill. 14(28):pii = 19269 19607781 10.2807/ese.14.28.19269-en

[21] Doherty L, McCartney M, Mitchell E, Wilson TS (1997) An outbreak of Salmonella enteritidis phage type 4 infection in a rural community in Northern Ireland. Commun Dis Rep CDR Rev. 5 2;7(5):R73–6. 9175310

[22] Cleary P, Browning L, Coia J, Cowden J, Fox A, Kearney J, et al; outbreak control team. (2010) A foodborne outbreak of Salmonella Bareilly in the United Kingdom, 2010. Euro Surveill. 12 2;15(48) 21144449 10.2807/ese.15.48.19732-en

[23] Buchholz U, Bernard H, Werber D, Böhmer M, Remschmidt C, Wilking H, et al (2011) German Outbreak of Escherichia coli O104:H4 Associated with Sprouts. NEJM. 11; 305(19) 10.1056/NEJMoa1106482 22029753

